# Construct Validity and Reliability of a New Basketball Multidirectional Reactive Repeated Sprint Test

**DOI:** 10.3390/ijerph182010695

**Published:** 2021-10-12

**Authors:** Seifeddine Brini, Daniel Boullosa, Julio Calleja-González, Anne Delextrat

**Affiliations:** 1 Research Unit, Sportive Performance and Physical Rehabilitation, High Institute of Sports and Physical Education of Kef, University of Jendouba, Kef 7100, Tunisia; 2 Faculty of Sciences of Bizerte, University of Carthage, Zarzouna, Bizerte 7021, Tunisia; 3 Integrated Institute of Health, Federal University of Mato Grosso do Sul, Campo Grande 79070-900, Brazil; daniel.boullosa@gmail.com; 4 College of Healthcare Sciences, James Cook University, Townsville 4811, Australia; 5 Physical Education and Sport Department, Faculty of Education and Sport, University of the Basque Country (UPV/EHU), 01007 Vitoria-Gasteiz, Spain; julio.calleja.gonzalez@gmail.com; 6 Department of Sport and Health Sciences and Social Work, Oxford Brookes University, Oxford OX3 0BP, UK; adelextrat@brookes.ac.uk

**Keywords:** performance, fatigue, agility, team sport, shuttle running

## Abstract

The objective of this study was to investigate the construct validity and reliability of a new reactive multidirectional repeated sprinting test (RRSA_5COD_) in basketball players. Forty male basketball players were divided into two groups: Professional (PRO; *n* = 20) and Semi-professional (SEMI; *n* = 20). Participants completed the yo-yo intermittent recovery test level 1 (Yo-YoIR1), the squat jump (SJ), the counter movement jump (CMJ), the single leg drop jump (DJ), the 20-m sprint test, the planed multidirectional repeated sprinting test (PRSA_5COD_), and the RRSA_5COD_ test. Reaction time (RT) and movement time (MT), total time (TT), best time (BT), and fatigue index (FI) were assessed. Heart rate (HR) was continuously recorded, while rating of perceived exertion (RPE) and blood lactate concentration (LA) were measured post-tests. The reliability of the RRSA_5COD_ test was also assessed between two attempts with one week between them. The RRSA_5COD_ results demonstrated to be reliable with most of the variables showing ICC > 0.80. BA Bonferroni post hoc revealed a significant better TT in favor of RRSA_5COD_ (*p* < 0.001; ES = 0.15; small), and in favor of PRO (*p* < 0.001; ES = 0.006; small). The result showed a significant better performance in favor of PRO in all physical fitness tests. In conclusion, it was found that the RRSA_5COD_ discriminates between professional and semi-professional male basketball players, and the results were demonstrated to be reliable.

## 1. Introduction

Basketball is an intermittent team sport in which brief high-intensity efforts are interspersed with low-intensity recovery periods [1]. Given the relatively small court area compared to other outdoor team sports, it has been shown that successful basketball performance relies on physical capacities such as acceleration and changes of direction (COD) rather than speed [2].

Classically, repeated COD ability has been evaluated in basketball with shuttle tests with long distances (15 m) and a single 180° turn [3] and, more recently, with multiple shorter runs (5 m) with several 180° turns [4]. However, 180° turns over longer distances are only relevant to a few sequences during basketball games, such as rapid transitions from offence to defense. In this regard, a new repeated multidirectional sprint test following a T shape with five COD (three 180° degrees and two 90° degrees) has been recently validated [5] and subsequently used in recent applied research [6,7]. Interestingly, Buchheit et al. [8] previously reported that the specific angle of a COD can influence the neuromuscular and metabolic demands, with 180° being more metabolically demanding than a straight line, while 90° had lower energy demands [8]. From these previous studies, it can be suggested that more variants (more 90 degree turns, different shape, different angles, etc.) of this repeated five COD test would perhaps better replicate the varied neuromuscular and metabolic demands of basketball games.

On the other hand, one crucial element that these repeated COD tests did not take into account is the perceptive component of most actions in basketball. Players often initiate actions in response to stimuli from teammates or defenders, and this aspect of performance, which is referred to as reactive agility, can be also assessed with various previous protocols [4,9]. However, to our knowledge, after a review the literature, there is no basketball-specific test that analyzes the repeated nature of COD with a reactive component. In fact, there is only one validated test of reactive repeated sprints with COD developed specifically for soccer discussed in the literature [10].

In order to monitor players at different times of the season and evaluate the effectiveness of various training programs, a test needs to be reliable and has good construct validity (i.e., be able to discriminate players of different levels). It is also essential to investigate its performance determinants to find out if it assesses different abilities compared to a repeated planned agility test [11]. In this context, the only published reactive repeated sprint tests showed several correlations with endurance capacity, single planned agility, lower limb power, and sprint capacity [10]. However, the application of this test to basketball is limited by the fact that it has very different movement patterns and distances.

Considering a single reactive agility test in basketball, Scanlan et al. [4], reported that cognitive measures such as response and decision-making times had a greater influence than physical measures on performance in male basketball players. However, it is unknown if this influence would remain in a test with repeated sprints, as the factors influencing the first and subsequent sprints may be different [8]. Therefore, the main aims of the present study were to assess the construct validity, the reliability, and the determinants of performance of a new proposed multidirectional reactive repeated sprint test in male basketball players.

## 2. Materials and Methods

### 2.1. Participants

Forty male basketball players from two different teams playing in the first and second Tunisian basketball leagues volunteered for this study at the moment of testing. The sample size was justified by a priori power analysis in G*power software (Version 3.1.9.2; Universität Kiel, Kiel, Germany). Participants were recruited if they were currently playing basketball at either a professional or semiprofessional level, had a history of physical activity (≥three times per week) extending over the previous six months, had played basketball for at least six years, and none of them had any injury during the last three months before the experiments. A medical examination was performed by the team’s medical doctor before the study began in order to verify that all participants did not have any medical condition precluding their participation. In addition, no player was taking medications, drugs, other stimulants or ergo-nutritional supplements that could affect the parameters analyzed during the period of the study. Additionally, to define the differences between performance levels, the players were divided into two category level groups (see Table 1): professional (PRO; n = 20) [ currently playing in the first basketball league and had already played for the Tunisian national team] and semi-professional (SEMI; n = 20) [currently playing in the second basketball league and had never played for the Tunisian national team ], with each group including four guards, four shooting guards, four small forwards, four forwards, and four centers. The training experience was significantly different between groups (12.55 ± 1.47 and 11.20 ± 1.28 years for PRO and SEMI respectively (*p* < 0.05)). Their weekly practice load was ≈ 12 h and ≈ 8 h for PRO and SEMI respectively.

The experimental procedures, associated risks, and benefits were explained to all participants who signed a written consent form prior to participation. The study was approved by a local research ethics committee (approval No. 9/2019). The protocol was conducted according to the Declaration of Helsinki [12].

### 2.2. Procedures

**Figure 1:**
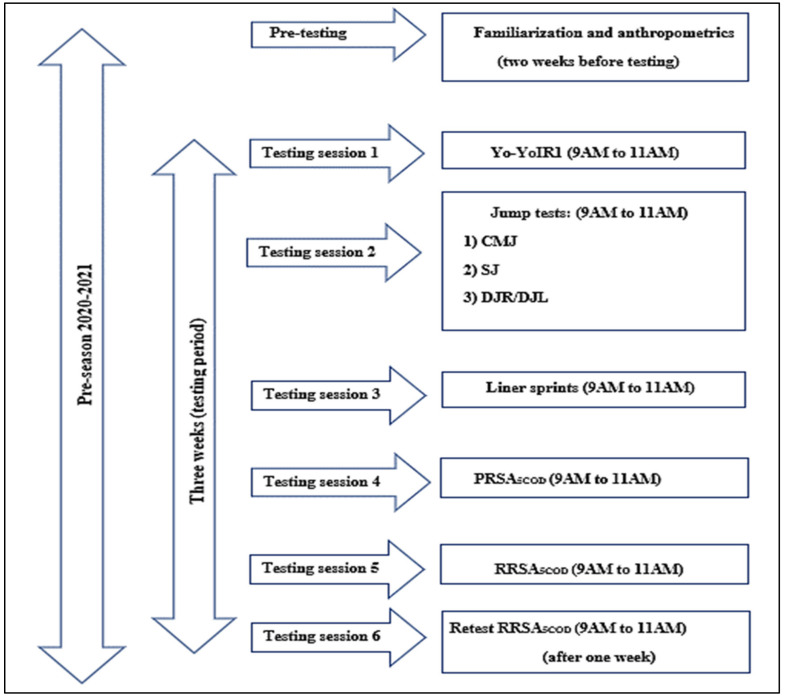
Experimental protocol. Legend: SJ: squad jump test, CMJ: countermovement jump test, DJR: single leg drop jump test (**right**), DRL; single leg drop jump (**left**); Yo-Yo IR1: Yo-Yo intermittent recovery test level 1.

Following pre-testing familiarization and anthropometric measurements which were conducted two weeks before, participants took part in six testing sessions. Sessions one to five were dedicated to testing the determinants of performance in the multidirectional reactive repeated sprint test (RRSA_5COD_), while the last session was a retest of session five to test the reliability of this test (see Figure 1). The study lasted three weeks and was conducted during the pre-season of the 2020/2021 season.

Testing sessions were separated by low intensity training sessions (shooting/tactical) with 48 h of rest before the Yo-Yo intermittent recovery test level one and a period of 48 h between (planned/reactive) sessions. To minimize any effects of circadian rhythms, all the testing sessions were conducted within ~2 h of the same time of day (during the morning). Players were instructed to wear the same footwear during all sessions and instructed to maintain consistent dietary and sleeping patterns during the testing period. Participants were also instructed to drink water “ad libitum” an hour before testing to ensure a euhydrated state. All testing sessions were conducted on a basketball court (wooden surface) in similar environments (temperature: 25.2 ± 3.9 °C; relative humidity: 61.1 ± 11.3%; barometric pressure: 757 ± 3 mmHg). All the team staff was always present during all testing and provided standardized verbal encouragement to all players. A 15-min standardized warm-up consisting of low-intensity jogging (8 min), whole body dynamic stretches (5 min), and brief bouts of high-intensity linear running (3 min) was prescribed to all participants before physical evaluations.

### 2.3. Assessment

#### 2.3.1. Pre-Testing: Familiarization and Anthropometrics

##### Familiarization Session

The participants underwent three familiarization trials for each test in the study. The first author (who is a certified strength and conditioning coach) demonstrated the proper form for the execution of all jumping tests and COD repeated sprinting tests. Previous studies within the field have reported that familiarization is a crucial component as athletes typically find a preferable movement repertoire that enables them to achieve their best result [13].

##### Anthropometrics

The body mass was evaluated in kilograms with a precision of 0.1 kg using an electronic balance (Pharo 200 Analytic, Germany) which was calibrated regularly. It was recommended to be presented without shoes and with light clothes. Height was measured in centimeters with a precision of 0.1 cm using a portable stadiometer (Seca, Maresten, UK), the body mass index (BMI) was then determined by dividing the weight (kg) by the square of the height (m). The body fat percentage was determined by the four skin folds method (biceps, triceps, subscapular, and suprailiac skinfolds) using pliers (Harpenden caliper). The fat percentage: BF% = (4.95/Body density − 4.5) × 100); (Body density = 1.162 2 0.063 3 log the sum of four skin folds) [14]. Anthropometric measurements were taken according to the recommendations from the International Society for the Advancement of Kinanthropometry (ISAK). All these measures were taken by a qualified technician.

#### 2.3.2. Yo-Yo Intermittent Recovery Test Level 1

The yo-yo intermittent recovery test level 1 (Yo-Yo IR1) was used to assess aerobic-anaerobic capacity. The test consisted of 20 m shuttle runs performed at increasing velocities with 10 s of active recovery between the shuttle runs, until volitional exhaustion [15]. Audio cues of the yo-yo IR1 test were played on a CD player (Philips, Az1030 CD player, Eindhoven, Holland). The end of the test was considered when the participant failed twice to reach the front-line on time or was unable to complete another shuttle at the dictated speed. The total distance (TD) covered during the Yo-Yo IR1 was recorded for further comparisons.

#### 2.3.3. Jump Tests

Participants performed three different jump tests in the following order (counter movement jump (CMJ), squat jump (SJ) and single leg drop jump (DJ) (right/left)). All jumps were performed three times with 2 min of rest between the attempts. The best score was used for the analysis. Vertical jump height was evaluated using an optoelectrical system (Opto-Jump Microgate, Italy). Jump height was calculated according to the following equation: jump height = 1/8 × g × t 2, where g is the acceleration due to gravity and t is the flight time [16]. CMJ and the SJ were performed according to previously described protocols [17]. To assess interlimb asymmetry, a drop jump with one leg was also performed. At the evaluator’s command, the participants started the movement standing upon the top of a 30 cm box. The asymmetry index used the following formula: (Highest performing limb-Lowest performing limb/Highest performing limb) ×100 [18].

#### 2.3.4. Linear Sprints

A 20-m sprint test was used to measure the acceleration and speed qualities. The participants stood 1 m behind the start line in a middle stance starting position with the body leaned forward. The time was recorded with photocells with an accuracy of 1 milisecond (Brower timing system, Salt Lake City, UT, USA) placed on the start (0 m) and finish (20 m) lines, with reflectors at 1 m. The participants performed three attempts with a 2-min rest period between each sprint. The best score was used for the analyses.

#### 2.3.5. Planned Repeated Multidirectional Sprint Test

Planned repeated multidirectional sprint tests (PRSA_5COD_) was performed. This test has been previously validated [5] and consisted of 10 × 30-m shuttle sprints following a T shape, with three changes of direction of 180° and two changes of direction of 90°, separated by 30 s of recovery. The COD occurred after each 5-m of running. The fatigue index (FI) was calculated using the Fitzsimons [19] formula: (100 × (TT/ (BT × 10)) − 100), where TT corresponds to total time (s) and BT to best time (s). The time for each attempt was recorded with photocells with an accuracy of 1 m (Brower timing system, Salt Lake City, UT, USA).

#### 2.3.6. Reactive Repeated Multidirectional Sprint Test

**Figure 2:**
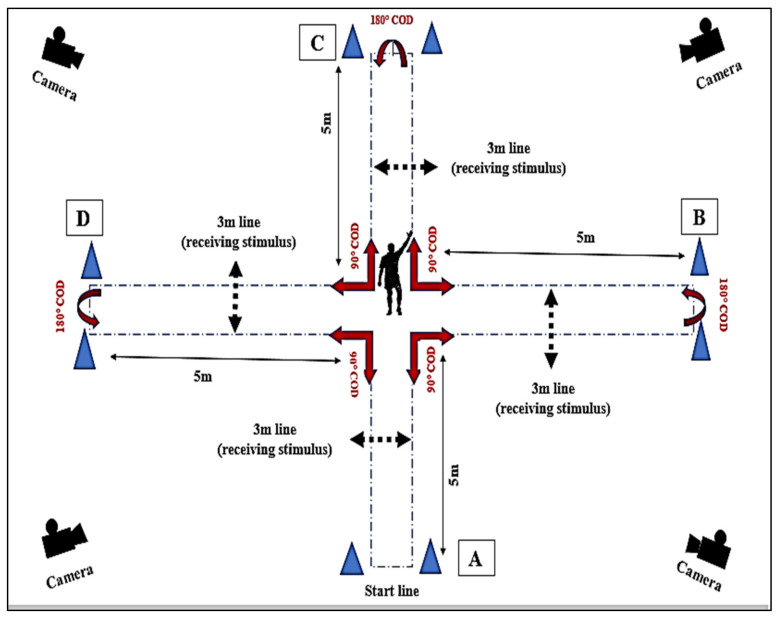
Graphical representation of the reactive multi change of direction (RRSA_5COD_) test.

-Design and equipment: The new Multidirectional Reactive Repeated Sprint Test (RRSA_5OD_) consist of a cross shape. The design and the dimensions of the newly developed test are shown in Figure 2. Four cameras (Handycam DCR-SR21; Sony, Tokyo, Japan) were placed around the testing area to obtain video data for each sprint realized during the test (i.e., the distance between each camera and the center of the cross is 8 m). The performance measures were recorded with a frequency of 60 Hz and subsequently calculated with the Kinovea software (v.0.8.15, France) [20].

-Test procedure: In general, the participants performed ten repetitions of [the sum of short sprint (5 m + 5 m + 5 m + 5 m + 5 m + 5 m) with either 180° COD or (90° COD following visual stimulus given by the tester)] with 30 s of recovery between repetitions. The changes of directions occurred each 5-m of running, either (180°) or (90°) in response to a visual stimulus performed by the tester indicating the side of the rotation. The tester was positioned in the center of the cross (30 cm behind the central cone) and always facing the participant (i.e., in front of the starting point or after each 180°COD). The tester gave visual stimulus (i.e., raising the right or left hand) to show the direction when the participant reached the (3 m) line in every 5 m sprint towards the center of the cross, while the participant continued the sprint (~2m), to finally complete a change of direction of 90° to mirror the direction showed by the tester (see Figure 2).

-Example for one repetition: [The participant commenced from the (A: Start line); sprinting 5 m to the center of the cross (in the meantime receiving visual stimulus at 3 m line); performing 90° COD; sprinting 5 m to (line B: performing 180° COD); Sprinting 5 m to the center of the cross (in the meantime receiving visual stimulus at 3 m line); performing 90° COD; sprinting 5 m to (line C: performing 180° COD); Sprinting 5 m (in the meantime receiving visual stimulus at 3 m line); performing 90° COD; sprinting 5 m to (line D: Finish line)].

** NB: Following the design and the procedure of the RRSA_5COD_, the participant may start and finish the test from any point (A, B, C or D) provided he runs a total distance of 30 m for each repetition.

-Test parameters:

* Reaction time (RT) was calculated as the duration from movement initiation of the tester until the first participant’s foot movement detected near the 3-m line point.

* Total reaction time (TRT) = (three RT in 30 m) × 10 repetitions and best reaction time (BRT) were recorded.

* Movement time (MT) was taken as the time interval from movement initiation of the tester until the first 180° COD line point reached by the participant.

* Total movement time (TMT) = (three MT in 30 m) × 10 and the best movement times (BMT) were also recorded.

* The fatigue index (FI) was calculated following previously described methods [19]. TT corresponds to total time and BT to best time.

#### 2.3.7. Heart Rate Measures

The heart rate (HR) during PRSA_5COD_ and RRSA_5COD_ tests was recorded continuously using a validated monitor (Polar Team2 Pro System, Polar Electro OY, Kempele, Finland), on a beat-to-beat.

#### 2.3.8. Session Rating of Perceived Exertion

The rating of perceived exertion (RPE) was recorded to determine the RRSA_5COD_ and PRSA_5COD_ working loads after the end of the testing protocols with the Borg’s CR-10 scale [21]. Players were largely familiarized with this procedure, as it was regularly used during their practices.

#### 2.3.9. Blood Lactate Concentration

Blood lactate concentration (LA) (mmol·L^−1^) was measured from capillary blood samples obtained from the earlobe at the third minute after the end of the RRSA_5COD_ and PRSA_5COD_ tests [22], using a portable lactate analyzer (Arkray Lactate Pro LT-1710 Kyoto, Japan), which was previously calibrated following the manufacturer’s instructions [23].

### 2.4. Statistical Analyses

All the data were expressed as Mean ± SD. The Shapiro Wilk Test (<50) identified all variables as normally distributed. The homoscedasticity of the variables was tested with Levene’s test. Relative reliability was established by calculating intra-class coefficients (ICC, model 3,1), while absolute reliability was assessed by the standard error of measurement (SEM) and coefficient of variation CV% = (SEM/Mean) × 100 [24,25]. The smallest worthwhile change (SWC) was computed as 0.3 of the between-subjects SD. Differences between tests (PRSA_5COD_ and RRSA_5COD_) and players levels (PRO and SEMI) were evaluated by an analyses of variance (ANOVA) mixed model. A Bonferroni post hoc test was used for identifying pairwise differences when a significant interaction was detected. Within-group ES were computed using the following equation: ES = (mean post–mean pre)/SD [26]. In accordance with Hopkins et al. [27], ES were considered trivial (<0.2), small (0.2–0.6), moderate (0.6–1.2), large (1.2–2.0), and very large (2.0–4.0). ANOVA was used to assess differences in physical fitness test performances. A stepwise regression was applied with BT as the dependent variable, and with all other parameters as independent variables. The correlation coefficients (r) were interpreted in accordance with the following scale of magnitude: trivial (r ≤ 0.1), small (r > 0.1–0.3), moderate (r > 0.3–0.5), large (r > 0.5–0.7), very large (r > 0.7–0.9), nearly perfect (r > 0.9), and perfect (r = 1.0). All statistical analyses were computed using SPSS for Windows, version 20 (SPSS Inc., Chicago, IL, USA). The level of significance was set at *p* < 0.05.

## 3. Results

All players from both experimental groups completed the study according to the previously described study design and methodology. No injuries related to training or testing occurred over the course of the experimental period.

Table 2 shows the reliability of RRSA_5COD_. In general, the ICCs ranged from appropriate to high values (0.80–0.99), with the strongest reliability evidenced for BT, TMT and HR. Moreover, the highest CV was evidenced for the Lac (5.63%) and the lowest was recorded for the TT (0.19%).

Table 3 shows the performances and physiological responses during the RRSA_5COD_ and PRSA_5COD_ tests. A significant main effect of test, group and interaction (group × test) for TT was observed (*p* < 0.001). Bonferroni post hoc revealed a significantly better TT in favor of RRSA_5COD_ (between tests) (*p* < 0.001; ES = 0.15; small) and for PRO (between groups) (*p* < 0.001; ES = 0.006; small). Also, significant main effects of test and group were observed for BT (*p* < 0.001). A significant main effect of group only was observed for FI (*p* < 0.001). Concerning physiological parameters, only a significant effect of group for HR and LA was recorded (*p* < 0.05). Moreover, significant main effects of test and group for RPE was observed (*p* < 0.05).

Reaction and movement times during RRSA_5COD_ are presented in Table 4. No significant differences were recorded between groups (PRO vs. SEMI) concerning BRT and TRT (*p* > 0.05) However, comparison between the groups revealed a significant faster BMT and TMT in favor of PRO (*p* < 0.001).

Table 5 illustrates the findings for jump, sprint and yo-yoIR1 performances determined for PRO and SEMI. The result showed a significantly better performance in favor of PRO in comparison with SEMI for all those parameters for CMJ; SJ; DJR; DJL; 20 m sprint and YoYoIR1 (*p* < 0.001).

Table 6 presented the correlations between RRSA_5COD_ test parameters, physical fitness tests and anthropometric parameters. A significant correlation was found among RRSA_5COD_ test parameters (dependent) [BT; FI; BMT; TMT; HR] and (independent) [physical fitness tests and anthropometric parameters] (see Table 6).

The stepwise multiple linear regression model for RRSA_5COD_ and PRASA_5COD_ selected only Yo-Yo IR1 and SJ as predictors of BT, with a [R^2^ = 0.46 (std. ß =0.000188; *p* = 0.004; std. ß = 0.007; *p* = 0.004; for Yo-Yo IR1 and SJ, respectively) and R^2^ = 0.35 (std. ß = 0.000211; *p* = 0.025; std. ß = 0.008; *p* = 0.015; for Yo-Yo IR1 and SJ, respectively)].

**Figure 3:**
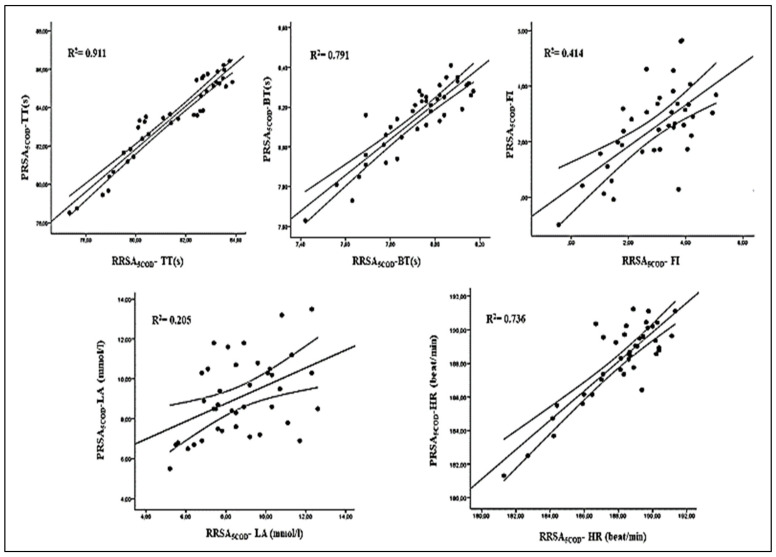
Correlation between RRSA_5COD_ and PRSA_5COD_ tests for professional and semi-professional group for each parameter. Legend: TT: total time; BT: best time; FI: fatigue index; HR: heart rate; LA: Lactate concentration. For each graph is reported the regression line and the 95% confidence interval.

Figure 3 presents the correlations between the RRSA_5COD_ and PRSA_5COD_ tests for each parameter. The results showed that, except for the RPE, all the variable of the two tests were significantly correlated (*p* < 0.05). (See Figure 3).

## 4. Discussion

The main objective of this study was to investigate the reliability and construct validity of a new reactive multidirectional repeated sprinting test to discriminate between the performance level of male senior basketball players. Our results showed that the newly developed RRSA_5COD_ was characterized by good reliability. In addition, it successfully discriminated professional and semi-professional basketball players, with better results observed in professionals. Moreover, the stepwise multiple linear regression model selected only Yo-Yo IR1 and SJ as determinants of performance for RRSA_5COD_.

### 4.1. RRSA_5COD_ Test Reliability

Most of the RRSA_5COD_ test variables showed a good test-retest reliability in terms of ICC (>0.80; see Table 2), and all of them showed small SEMs (see Table 2). These results were similar to previous studies [5,28,29] which showed good test-retest reliability of the COD tests and RSA outcomes during PRSA_5COD_, respectively (ICC > 0.85). Additionally, our findings revealed a good test-retest reliability in terms of ICC, CV and SEM when compared with previous studies examining different reactive COD tests [11,26]. In general, those tests varied in their reliability, with ICCs that ranged from 0.84 to 0.99 and 0.81 to 0.88 for the pre-planned vs. non planned tests, respectively [30].

Our findings revealed that, in the majority of RRSA_5COD_ test parameters, SEM ≤ SWC, which indicated the test capacity to detect changes after any intervention. This suggests very good sensitivity for measuring changes in physical performance, which was similar to the study by Di Mascio et al. [10]. In this context, TT was the parameter with the better CV compared to BT and FI (CV: 0.19 vs. 0.36 vs. 0.99%). Moreover, the CV found in this study for the TT was lower to the values reported by Fitzsimons [19]. Impellizzeri et al. [31] and Di Mascio et al. [10] reported a CV of 0.8% for the total and average time, respectively. This lower CV value could be explained by the differences in test design (which do not reflect the protocol of our RRSA_5COD_ test) and studies populations (basketball players vs. soccer players). Furthermore, the CV (0.36%) of the BT recorded in our investigation was lower than the CV reported by Di Mascio et al. [10] (1.04%) and Impellizzeri et al. [31] (1.3%). Moreover, The BT was the parameter with the higher ICC in our investigation when compared to TT and FI (ICC: 0.98 vs. 0.95 vs. 0.83).

FI was the parameter with the lower reliability in comparison with TT and BT. Furthermore, the SEM > SWC indicates that this parameter is rated as “marginal”. In this context, previous studies reported the low reliability of FI during multiple sprint bouts among active men and the difficulty in practically interpreting FI, therefore the usefulness of this index is still up for debate [32]. Our findings showed a good test retest for HR mean, LA and RPE (ICC: 0.99, 0.95, 0.80, respectively). For LA, the ICC was higher than the value reported in the study by Zagatto et al. [5] (ICC: 0.95 vs. 0.74, respectively). However, we recorded a lower ICC for RPE in comparison with Padulo et al. [29] (ICC: 0.80 vs. 0.91, respectively). Finally, adding the reactive component to our test has probably decreased the reliability, especially in comparison with single agility testing or PRSA_5COD_ but, overall, our reliability is good and all these results confirm the reliability of the RRSA_5COD_ test.

### 4.2. Construct Validity (Performance Level Differences, PRO vs. SEMI)

One of the prerequisites of test applicability in sport science is its construct validity [28]. Construct validity is commonly established by testing differences among groups of participants of different competitive levels [28]. Previous studies reported that reactive COD testing has confirmed that higher skilled players have a superior ability to extract and utilize advanced cues from opponents more quickly than their lesser skilled peers, which supports our results [30]. In fact, the most important findings of the present study were that the new RRSA_5COD_ test discriminates well between players’ levels.

Our results showed that PRO players achieved significantly better performances than SEMI (especially for TT and BT). Moreover, differences in TT, BT and FI performances between PRO and SEMI are very similar for both RRSA_5COD_ and PRSA_5COD_. Our findings could be justified by the fact that both tests have the same determinants of performance (Yo-YoIR1 and SJ), this could explain that PRO are better than SEMI, as we know they have better aerobic fitness (Yo-YoIR1), strength (SJ) and a better training status (i.e., full-time players). Furthermore, basketball-specific playing duties in a higher level often challenge different facets of agility [4]. Moreover, the significant differences in the RRSA_5COD_ between performance levels could be also explained by the fact that PRO players in our investigation possessed a significant lower BMI and BF% in comparison with SEMI players. In this context, previous studies reported that some parameters, such as the body dimensions and BF%, can be considered as important determinants of change of direction ability in reactive-agility [13]. Concerning RT during the RRSA_5COD_ test, we did not observe a significant difference between groups. It was expected that the perception-action coupling would represent the cognitive determinant of agility. However, the cognitive part of agility performance is minor given that it lasts only a fraction of a second. The essential determinant is the physical determinant (COD ability), given that it takes up the main part of the entire task [33].

### 4.3. Effects of RRSA_5COD_ versus PRSA_5COD_ Tests on RSA Outcomes

Our findings showed that the RRSA_5COD_ test led to a significantly better TT and BT for both groups in comparison with the PRSA_5COD_ test. Those results could be mainly explained by the differences in the protocol design between the two tests. In fact, RRSA_5COD_ was designed with more CODs of 90° and less CODs of 180° when compared to the PRSA_5COD_ test. Moreover, comparisons with previous studies investigating RSA tests including one or two CODs revealed that RRSA_5COD_ performance time seems to be slower, which is in agreement with previous findings [29,34]. Concerning physiological parameters, the two tests were not statistically different from each other in terms of HR responses and LA. This suggests that, despite faster sprint times in the RRSA_5COD_ test compared to PRSA_5COD_, the two tests were performed under similar metabolic conditions. Furthermore, our results showed that RRSA_5COD_ leads to higher physiological demands, which could represent an efficient mean to increase physiological markers of fatigue, as supported by the RPE values recorded in our study. Additionally, our results revealed that the majority of the RRSA_5COD_ parameters, such as TT, BT, FI, HR and LA, were significantly correlated to those recorded for PRSA_5COD_. Furthermore, our findings showed that only Yo-Yo IR1 and SJ were performance predictors for both RRSA_5COD_ and PRSA_5COD._ However, despite the similarity and the high correlations between the two tests, especially in physiological parameters such as (HR and LA), and with respect to the PRSA_5COD,_ we advise coaches and physical trainers in the basketball field to use the RRSA_5COD_ because this newly developed test is able to measure both the physical and cognitive factors and have a more specific design. In this context, previous studies suggested the inclusion of some type of perceptual and decision-making component in order to thoroughly distinguish COD performance between basketball players of different ability levels [4,11].

Finally, our study presented some limitations. RRSA_5COD_ was tested by using a test in which players responded to a visual stimulus in which the choice of direction was limited to only two (i.e., left or right) options. Secondly, our study was aimed to evaluate a reactive multidirectional repeated sprint without a ball, while in real basketball game might include some sequences in which some players’ position (i.e. guards) could replicate those specific movements with the ball. Thus, further studies should take that approach into consideration in order to improve predictions of the performance of basketball players.

## 5. Conclusions

Our results showed that the RRSA_5COD_ is highly reliable and allows the differentiation between professional and semiprofessional basketball players. Moreover, the majority of the RRSA_5COD_ parameters were correlated to previously validated PRSA_5COD_ tests in basketball. Thus, it can be concluded that the RRSA_5COD_ is valid and reliable for basketball players, and this newly developed COD repeated sprint test is recommended for further use by strength and conditioning professionals, sport scientists and practitioners.

### Practical Applications

Our findings provide novel insight into the influence of both physical and cognitive factors on agility performance using open-skill assessments of players’ directional changes in response to external stimuli during training and match-play. COD ability has the greatest influence on reactive multidirectional repeated sprints in basketball players.

## Figures and Tables

**Table 1:** Characteristics of the basketball players.

Basketball Players	Age (Years)	Height (cm)	Body Mass (kg)	BMI (kg.m^−2^)	BF%
PRO (n = 20)	25.75 ± 2.84	1.93 ± 0.08	86.80 ± 6.79	23.22 ± 1.24	10.75 ± 2.24
SEMI (n = 20)	27.35 ± 3.27	1.91± 0.09	88.35 ± 9.70	24.16 ± 1.05	14 ± 2.47

Legend: data are means and standard deviations. PRO: Professional group; SEMI: semi-professional group; BMI: Body mass index; BF: Body fat.

**Table 2:** Test/re-test reliability of the reactive multidirectional repeated sprints test.

Variables		RRSA_5COD_				
		ICC	95% CI	%CV	SEM	SWC
TT		0.95	0.91–0.98	0.19	0.43	0.58
BT		0.98	0.89–0.99	0.36	0.02	0.05
FI		0.83	0.69–0.91	0.99	0.55	0.40
RT	BRT	0.89	0.78–0.94	3.78	0.01	0.01
	TRT	0.98	0.97–0.99	0.49	0.10	0.21
MT	BMT	0.98	0.94–0.99	1.03	0.02	0.04
	TMT	0.99	0.60–0.99	0.43	0.37	1.10
HR		0.99	0.83–0.99	0.06	0.22	0.67
LA		0.95	0.89–0.97	5.63	0.39	0.52
RPE		0.80	0.62–0.89	2.34	0.23	0.16

Legend: Test/re-test reliability between measures for RRSA_5COD_. TT: total time; BT: best time; FI: fatigue index; HR: heart rate; LA: Lactate concentration; RPE: rating of perceived exertion RT, Reaction time; BRT, Best reaction time; TRT, Total reaction time; MT, movement time; BMT, Best movement time; TMT, Total movement time. The reliability is expressed both with the Intra-class Correlation Coefficient (ICC) and the Standard Error of the Mean (SEM).

**Table 3:** RSA Time performances and physiological parameters during RRSA_5COD_ and PRSA_5COD_ tests for professional and semi-professional group.

							*p*-Values (Effect Size)		
Variables	RRSA_5COD_			PRSA _5COD_			Test	Group	Group × Test
	PRO	SEMI	Δ%	PRO	SEMI	Δ%			
TT (s)	81.32 ± 1.86 ^£^	81.57 ± 1.87	−0.31 ± 0.04	83.35 ± 2.19	83.56 ± 2.17	−0.26 ± 0.05	0.000 (0.90)	0.000 (0.99)	0.010 (0.30)
BT (s)	7.83 ± 0.17 ^£^	7.99 ± 0.15	−1.99 ± 0.96	8.07 ± 0.17	8.21 ± 0.16	−1.72 ± 1.08	0.000 (0.91)	0.000 (0.84)	0.399 (0.04)
FI (%)	3.77 ± 0.58 ^£^	2.05 ± 1.18	46.74 ± 28.92	3.25 ± 1.07	1.77 ± 1.44	48.74 ± 33.90	0.094 (0.14)	0.000 (0.80)	0.389 (0.03)
HR (beat/min)	186.90 ± 2.46 ^†^	189.28 ± 1.25	−1.29 ± 1.58	186.93 ± 2.45	189.45 ± 1.28	−1.37 ± 1.58	0.640 (0.01)	0.001 (0.45)	0.692 (0.008)
LA (mmol/L)	7.99 ± 1.97 *	9.46 ± 1.68	−24.33 ± 34.94	8.45 ± 1.82	9.73 ± 1.86	−20.25 ± 34.13	0.297 (0.06)	0.009 (0.31)	0.763 (0.005)
RPE	6.75 ± 0.64	6.90 ± 0.64	−3.36 ± 15.49	7 ± 0.73	7.55 ± 0.51	−9.02 ± 14.26	0.004 (0.37)	0.049 (0.19)	0.163 (0.10)

Legend: Data are mean and standard deviations. ES, Effect size; TT: total time; BT: best time; FI: fatigue index; HR: heart rate; LA: Lactate concentration; RPE: rating of perceived exertion; * Significantly different between groups for RRSA_5COD_, *p* < 0.05; ^†^ Significantly different between groups for the RRSA_5COD_, *p* < 0.01; ^£^ Significantly different between groups for the RRSA_5COD_, *p* < 0.001.

**Table 4:** Reaction and movement times determined during the RRSA_5COD_ test for both groups.

Variables		PRO			SEMI		
		Mean	SD	95% IC	Mean	SD	95% IC
RT(s)	BRT(s)	0.34	0.03	0.32 to 0.35	0.35	0.02	0.34 to 0.36
	TRT(s)	10.40	0.48	10.01 to 10.80	10.69	0.51	10.45 to 10.92
MT(s)	BMT(s)	1.48 ***	0.10	1.43 to 1.53	1.67	0.07	1.63 to 1.70
	TMT(s)	45.29 ***	3.24	43.78 to 46.81	50.25	2.21	49.21 to 51.28

Legend: Data are means and standard deviations. Moreover, the number of participants and the 95% of confident interval (CI) of the following variables are reported: RT, Reaction time; BRT, Best reaction time; TRT, Total reaction time; MT, movement time; BMT, Best movement time; TMT, Total movement time. *** Significantly different between groups, *p* < 0.001.

**Table 5:** Jump, sprint and Yo-Yo intermittent recovery test level 1 performances determined for professional and semi-professional group.

Variables		PRO			SEMI		
		Mean	SD	95%IC	Mean	SD	95%IC
CMJ (cm)		36.70 ***	3.18	35.21 to 38.19	32.95	2.60	31.73 to 34.17
SJ (cm)		34.95 ***	3.03	33.53 to 36.37	31.60	2.91	30.24 to 32.96
DJ (cm)	RIGHT	15.30 ***	2.08	14.33 to 16.27	12.75	1.65	11.98 to 13.52
	LEFT	14.90 *	2.53	13.72 to 16.08	13.30	1.92	12.40 to 14.20
	ASYMMETRY INDEX	14.16	1.26	11.53 to 16.79	14.75	1.01	12.62 to 16.88
20 m SPRINT (s)		3.55 ***	0.09	3.51 to 3.60	3.70	0.04	3.68 to 3.72
YO-YO IR1 (m)		1987 ***	133.03	1924.74 to 2029.47	1860	71.96	1826.31 to 1893.68

Legend: Data are means and standard deviations. SJ: squad jump test, CMJ: countermovement jump test, DJ: drop jump test; Yo-Yo IR1: Yo-Yo intermittent recovery test level 1; * Significantly different between PRO and SEMI, *p* < 0.05; *** Significantly different between PRO and SEMI, *p* < 0.001.

**Table 6:** Correlations between RRSA_5COD_ test parameters (dependent), physical fitness tests and anthropometric parameters (independent).

Variables		CMJ	SJ	DJR	DJL	YOYOIR1	20 m-Sprint	BM	BMI	BF%
TT		−0.25	−0.28	−0.02	-0.20	−0.30	0.16	0.13	−0.19	0.07
BT		−0.53 ***	−0.57 ***	−0.33 *	−0.41 **	−0.56 ***	0.34 **	0.27	−0.007	0.36 *
FI		0.51 ***	0.53 ***	0.57 ***	0.38 *	0.48 **	−0.50 ***	0.25	−0.34 *	−0.54 ***
RT	BRT	−0.03	−0.06	−0.13	0.09	−0.13	0.16	-0.15	0.14	−0.03
	TRT	0.03	−0.04	−0.02	0.14	−0.19	0.10	−0.10	0.04	−0.04
MT	BMT	−0.60 ***	−0.62 ***	−0.52 ***	−0.44 *	−0.53 ***	0.67 ***	0.19	0.28	0.49 ***
	TMT	−0.58 ***	−0.61 ***	−0.49 ***	−0.44 *	−0.52 ***	0.64 ***	0.20	0.27	0.47 **
HR		−0.48 **	−0.43 **	−0.45 **	−0.30	−0.40 **	0.50 ***	0.02	0.20	0.27
LA		−0.16	0.10	−0.21	−0.06	0.02	0.33	−0.13	−0.15	−0.03
RPE		−0.07	−0.08	−0.16	−0.06	−0.03	0.21	0.01	0.24	0.12

Legend: TT: total time; BT: best time; FI: fatigue index; HR: heart rate; LA: Lactate concentration; RPE: rating of perceived exertion SJ: squad jump test, CMJ: countermovement jump test, DJ: drop jump test; Yo-Yo IR1: Yo-Yo intermittent recovery test level 1; RT, Reaction time; BRT, Best reaction time; TRT, Total reaction time; MT, movement time; BMT, Best movement time; TMT, Total movement time. *: *p* < 0.05; **: *p* < 0.01; ***: *p* < 0.001.

## Data Availability

The data presented in this study are available on request from the corresponding author. The data are not publicly available due to privacy reasons.

## References

[1] Torres-Ronda L., Ric A., Llabres-Torres I., de Las Heras B., Schelling I.D.A.X. (2016). Position-dependent cardiovascular response and time-motion analysis during training drills and friendly matches in elite male basketball players. J. Strength Cond. Res..

[2] Delextrat A., Cohen D.J. (2008). Physiological testing of basketball players: Toward a standard evaluation of anaerobic fitness. Strength Cond. Res..

[3] Castagna C., Abt G., Manzi V., Annino G., Padua E., D’Ottavio S. (2008). Effect of recovery mode on repeated sprint ability in young basketball players. Strength Cond. Res..

[4] Scanlan A., Humphries B., Tucker P.S., Dalbo V. (2014). The influence of physical and cognitive factors on reactive agility performance in men basketball players. J. Sports Sci..

[5] Zagatto A.M., Ardigo L.P., Barbieri F.A., Milioni F., Iacono A.D., Camargo B.H., Padulo J. (2017). Performance and metabolic demand of a new repeated-sprint ability test in basketball players: Does the number of changes of direction matter?. J. Strength Cond. Res..

[6] Brini S., Ben Abderrahman A., Boullosa D., Hackney A.C., Zagatto A.M., Castagna C., Bouassida A., Granacher U., Zouhal H. (2020). Effects of a 12-Week Change-of-Direction Sprints Training Program on Selected Physical and Physiological Parameters in Professional Basketball Male Players. Int. J. Environ. Res. Public Health.

[7] Brini S., Delextrat A., Bouassida A. (2021). Variation in lower limb power and three point shot performance following repeated sprints: One vs. five changes of direction in male basketball players. J. Hum. Kinet..

[8] Buchheit M., Haydar B., Ahmaidi S. (2012). Repeated sprints with directional changes: Do angles matter?. J. Sports Sci..

[9] Spiteri T., Newton R.U., Nimphius S. (2015). Neuromuscular strategies contributing to faster multidirectional agility performance. J. Electromyogr. Kinesiol..

[10] Di Mascio M., Ade J., Bradley P.S. (2015). The reliability, validity and sensitivity of a novel soccer-specific reactive repeated-sprint test (RRST). Eur. J. Appl. Physiol..

[11] Lockie R.G., Jeffriess M.D., McGann T.S., Callaghan S.J., Schultz A.B. (2014). Planned and reactive agility performance in semi-professional and amateur basketball players. Int. J. Sports Physiol. Perform..

[12] Hellmann F., Verdi M., Schlemper B.R., Caponi S. (2014). 50th anniversary of the Declaration of Helsinki: The double standard was introduced. Arch. Med. Res..

[13] Sekulic D., Spasic M., Mirkov D., Cavar M., Sattler T. (2013). Gender-specific influences of balance, speed, and power on agility performance. J. Strength Cond. Res..

[14] Sisic N., Jelicic M., Pehar M., Spasic M., Sekulic D. (2016). Agility performance in high-level junior basketball players: The predictive value of anthropometrics and power qualities. J. Sports Med. Phys. Fit..

[15] Castagna C., Impellizzeri F.M., Rampinini E., D’Ottavio S., Manzi V. (2008). The Yo-Yo intermittent recovery test in basketball players. J. Sci. Med. Sport..

[16] Prieske O., Muehlbauer T., Mueller S., Krueger T., Kibele A., Behm D., Granacher U. (2013). Effects of surface instability on neuromuscular performance during drop jumps and landings. Eur. J. Appl. Physiol..

[17] Bosco C., Luhtanen P., Komi P. (1983). A simple method for measurement of mechanical power in jumping. Eur. J. Appl. Physiol..

[18] Impellizzeri F.M., Rampinini E., Maffiuletti N., Marcora S.M. (2007). A vertical jump force test for assessing bilateral strength asymmetry in athletes. Med. Sci. Sports Exerc..

[19] Fitzsimons M., Dawson B., Ward D., Wilkinson A. (1993). Cycling and running tests of repeated sprint ability. Aust. J. Sci. Med. Sport..

[20] Albert P., Carles E.M., Josep Maria P.R., Albert B., Xavier P.C., Daniel M.R. (2019). Validity and Reliability of the Kinovea Program in Obtaining Angles and Distances Using Coordinates in 4 Perspectives. PLoS ONE.

[21] Foster C. (1998). Monitoring training in athletes with reference to overtraining syndrome. Med. Sci. Sports Exerc..

[22] Hirvonen J., Rehunen S., Rusko H., Harkonen M. (1987). Breakdown of high-energy phosphate compounds and lactate accumulation during short supramaximal exercise. Eur. J. Appl. Physiol. Occup. Physiol..

[23] Iorizzo L., Klausen T.W., Wiberg-Itzel E., Ovin F., Wiberg N. (2019). Use of Lactate Pro (TM)2 for measurement of fetal scalp blood lactate during labor—Proposing new cutoffs for normality, preacidemia and acidemia: A cross-sectional study. J. Matern.-Fetal Neonatal Med..

[24] Hopkins W.G. (2000). Measures of reliability in sports medicine and science. Sports Med..

[25] Weir J.P. (2005). Quantifying test-retest reliability using the intra-class correlation coefficient and the SEM. J. Strength Cond. Res..

[26] Cohen J. (1973). Eta-squared and partial eta-squared in fixed factor ANOVA designs. Educ. Psychol. Meas..

[27] Hopkins W.G., Marshall S.W., Batterham A.M., Hanin J. (2009). Progressive statistics for studies in sports medicine and exercise science. Med. Sci. Sports Exerc..

[28] Hachana Y., Chaabène H., Ben Rajeb G., Khlifa R., Aouadi R., Chamari K., Gabbett T.J. (2014). Validity and Reliability of New Agility Test among Elite and Sub-elite under 14-Soccer Players. PLoS ONE.

[29] Padulo J., Laffaye G., Haddad M., Chaouachi A., Attene G., Migliaccio G.M., Chamari K., Pizzolato F. (2015). Repeated sprint ability in young basketball players: One vs. two changes of direction (Part 1). J. Sports Sci..

[30] Pojskic H., Åslin E., Krolo A., Jukic I., Uljevic O., Spasic M., Sekulic D. (2018). Importance of Reactive Agility and Change of Direction Speed in Differentiating Performance Levels in Junior Soccer Players: Reliability and Validity of Newly Developed Soccer-Specific Tests. Front. Physiol..

[31] Impellizzeri F.M., Rampinini E., Castagna C., Bishop D., Ferrari Bravo D., Tibaudi A., Wisloff U. (2008). Validity of a repeated-sprint test for football. Int. J. Sports Med..

[32] Oliver J.L. (2009). Is a fatigue index a worthwhile measure of repeated sprint ability?. J. Sci. Med. Sport..

[33] Zouhal H., Abderrahman A.B., Dupont G., Truptin P., Le Bris R., Le Postec E., Sghaeir Z., Brughelli M., Granacher U., Bideau B. (2019). Effects of Neuromuscular Training on Agility Performance in Elite Soccer Players. Front. Physiol..

[34] Attene G., Nikolaidis P.T., Bragazzi N.L., Dello Iacono A., Pizzolato F., Zagatto A.M., Dal Pupo J., Oggianu M., Migliaccio G.M., Mannucci Pacini E. (2016). Repeated Sprint Ability in Young Basketball Players (Part 2): The Chronic Effects of Multidirection and of One Change of Direction Are Comparable in Terms of Physiological and Performance Responses. Front. Physiol..

